# Developing easy access patient information booklets and consent forms for use in multicentre stroke trials

**DOI:** 10.1186/1745-6215-14-S1-P58

**Published:** 2013-11-29

**Authors:** Gillian Mead, Martin Dennis, Marian Brady

**Affiliations:** 1 University of Edinburgh, Edinburgh, UK; 2 Glasgow Caledonian University, Glasgow, UK

## 

Global cognitive impairment is a common consequence of stroke, and some stroke patients will have had dementia prior to their stroke. Thus, many people with acute stroke are often considered unable to consent to participation in research and proxy consent is standard. Aphasia is a common neurological consequence of stroke, affecting several aspects of language including speaking, writing, reading and understanding but not necessarily impacting on self determination. Ethically, patients with capacity to consent should be enabled to engage in the information provision and consent process rather than making an assumption of incapacity.

For our ongoing multicentre FOCUS trial (http://www.focustrial.org.uk), we developed ‘easy-access' patient information booklets and consent forms to facilitate information provision and informed consent with people with communication impairment.

Co-produced in an iterative process with stroke survivors with aphasia (Speakeasy, Dundee) the information provided in the standard forms was refined into a more accessible format. Short sentences, simple grammatical structures and large well spaced text was supplemented with photographs, where possible, to support the information provision process. Approved by Scotland A Research Ethics Committee, preliminary experience suggests the ‘easy-access' versions have supported patients with mild aphasia. Recording which version of the information booklets was used and the presence and severity of aphasia (National Institute of Health Stroke Scale) will enable us to report how many patients with aphasia were enabled to independently consent to participation in the FOCUS trial.

